# A broadly applicable COI primer pair and an efficient single‐tube amplicon library preparation protocol for metabarcoding

**DOI:** 10.1002/ece3.4520

**Published:** 2018-12-11

**Authors:** Oskar Rennstam Rubbmark, Daniela Sint, Nina Horngacher, Michael Traugott

**Affiliations:** ^1^ Mountain Agriculture Research Unit Institute of Ecology University of Innsbruck Innsbruck Austria

**Keywords:** diet analysis, DNA barcoding, environmental DNA, high‐throughput sequencing, next‐generation sequencing, primer bias

## Abstract

The nucleotide variation in the cytochrome c oxidase subunit I (COI) gene makes it ideal for assigning sequences to species. However, this variability also makes it difficult to design truly universal primers. Here, we present the forward primer “Sauron‐S878,” specifically designed to facilitate library preparation for metabarcoding. This primer is modified to improve the coverage of terrestrial species compared to the primer mCOIintF, optimized for aquatic systems, which raised the in silico coverage from 74.4% to 98.3% of available NCBI sequences (perfect match in 3′ region, up to three mismatches in remaining primer). When paired with the reverse primer “jgHCO2198” (fragment length ~313 bp), these primers amplified 98.4% of 255 tested DNA extracts from various taxa, which are better than many other common COI barcoding primers. Furthermore, a single‐tube protocol was developed, wherein these primers amplify the target gene, and attach MIDs and Illumina sequencing adapters in one reaction. This eliminates the need for re‐amplification or enzymatic ligation during library preparation while keeping the flexibility to modularly combine primers and MIDs. Using the single‐tube approach, three replicates of three mock samples were sequenced on a MiSeq platform with no adverse effects compared to commercial Nextera indexing kits. From this run, 75% of all included taxa could be recovered, with no considerable bias among taxonomic groups. Despite the fact that 98.4% of the extracts were confirmed to amplify in vitro, this number was lower than expected. A reason for this discrepancy was a clear link between the relative concentration of a specific DNA type in the template and the number of returned reads for this DNA. We would argue that such a bias may be especially problematic in metabarcoding where samples usually contain trace DNA in unknown amounts. However, how this affects the completeness of metabarcoding results has yet been poorly investigated.

## 1 INTRODUCTION

Metabarcoding is an easy to use and powerful method that increasingly is being employed to detect the presence of species in applications ranging from the analysis of community bulk samples (Ji et al., 2013; Yu et al., 2012) to biodiversity assessments from environmental DNA (Bohmann et al., 2014; Taberlet, Coissac, Hajibabaei, & Rieseberg, 2012; Thomsen & Willerslev, 2015) and studies of trophic interactions (De Barba et al., 2014; Deagle, Kirkwood, & Jarman, 2009; Pompanon et al., 2012; Valentini et al. 2009). It combines DNA‐based identification of species (barcoding) with next‐generation sequencing (NGS or high‐throughput sequencing—HTS) by using so‐called universal primers, usually targeting a specific group of interest in order to mass amplify DNA from collected samples containing mixes of DNA (Taberlet et al., 2012). Metabarcoding has considerable advantages over more traditional approaches, where taxonomic assignment is done morphologically. For example, environmental samples can be collected in a way that minimizes disturbances to sensitive ecosystems compared to more traditional sampling methods (De Barba et al. 2010). In addition, by using existing sequence databases for species identification, hard to come by taxonomic expertise can be reduced.

By convention, the most commonly used gene for barcoding of Metazoan diversity has so far been the mitochondrial cytochrome c oxidase subunit one (COI) gene. The main reason for this is that, even though other genes have been shown to work better to identify plants (rbcL, matK; CBOL Plant Working Group, 2009), fungi (ITS; Schoch et al., 2012) and bacteria (16S; Tringe & Hugenholtz, 2008), COI has usually been suitable in identifying most animals to species level (Hebert, Cywinska, & Ball, 2003). Because of this, it was selected as the target gene for the barcode of life initiative (BOLD), and so far, the number of animal species sequenced for this gene fragment (~2.3 million sequences from ~280,000 species in GenBank) is much greater than for other common barcoding genes such as 16S (~380,000 sequences from ~90,000 species) or 18S (~170,000 sequences from ~70,000 species). A reason for this is that these alternative genes generally offer lower taxonomic resolution, which provides a strong argument for why COI is a good candidate for metabarcoding of animals. Particularly, as even though alternative barcoding regions may be more suited for primer design, these will restrict scientists to treating individual taxa as observed operational taxonomic units (OTUs; Ji et al., 2013). This can have negative effects on the quality of results, especially when reference sequences are not available or when species cannot be distinguished based on the delivered sequence information (e.g., 18S). This will hamper species identification and make important characteristics such as species traits difficult or impossible to assign.

The suitability of COI for metabarcoding is thus high; however, it is also being questioned (Deagle, Jarman, Coissac, Pompanon, & Taberlet, 2014) due to the fact that it has been difficult to design truly universal primers for this gene (Geller, Meyer, Parker, & Hawk, 2013). The reason for this is that as a coding gene, it exhibits considerable variation in every 3rd base, which means that highly conserved regions are lacking (Hebert et al., 2003). In fact, it has been shown that most tested COI primers that claim to be universal are only marginally so and often fail to amplify many taxa (Clarke, Soubrier, Weyrich, & Cooper, 2014; Deagle et al., 2014; Elbrecht & Leese, 2015; Leray et al., 2013). This means that among species that could be identified and confirmed present in a sample, there may be an unknown range of false absences due to methodological error. For interpreting results, this then becomes problematic. On one hand, this may not be of concern when comparing structural differences between samples (beta diversity), but could become very problematic for comparing differences in alpha diversity (Clooney et al., 2016). So far, however, the majority of the testing done to identify primer bias has been based on small sets of species or sequences from mainly aquatic and invertebrate taxa. Consequently, even for many of the more commonly used metabarcoding primers, there is still no comprehensive knowledge on their taxonomic coverage and for which taxa they work well or poorly.

For metabarcoding, it is furthermore especially important that this testing is done, not only by comparing the presence or absence of an amplification in vitro, but also by taking into account the differences in match and/or amplification efficiency between primers and DNA sequences. This is needed because, even when primers amplify the DNA of a certain species, if the fit of primers is different between taxa, the better fitting taxa will be amplified preferentially in a competitive reaction (Bru, Martin‐Laurent, & Philippot, 2008; Green, Venkatramanan, & Naqib, 2015). In mixed samples, this will be further problematic as such biases will interact with, for example, unequal amounts of biological material (e.g., tissue) or different copy numbers of the targeted gene depending on tissue type or species (Pompanon et al., 2012) and further affect the probability to detect a species. While there are attempts to develop PCR‐free approaches that are also suitable for metabarcoding (Creer et al., 2016; Denver, Brown, Howe, Peetz, & Zasada, 2016; Paula et al., 2016), currently there are still too many limitations to replace the so far used target sequencing in the near future.

Still, these different sources of bias are troubling, and solutions have, for example, included the design of primers that amplify less variable barcoding genes (e.g., “ribosomal markers” Clarke et al., 2014; Deagle et al., 2014). However, by using barcoding regions with less variability, taxonomic resolution will decrease and therefore it is as mentioned, desirable to attempt to reduce these biases in order to retain the use of COI. Fortunately, a number of recent studies have shown that while COI is variable, with some of the degenerate primers available such as mCOIintF (Leray et al., 2013) and jgHCO2198 (Geller et al., 2013), it is nevertheless possible to get at least for some groups a comparable taxonomic coverage to alternative barcoding regions (Clarke, Beard, Swadling, & Deagle, 2017; Elbrecht et al., 2016). The variation within COI does, however, mean that a large amount of degeneracy is needed. This is because even single mismatches close to the 3′ end of primers can significantly reduce the amplification efficiency of a given taxon in a PCR (Green et al., 2015; Lefever, Pattyn, Hellemans, & Vandesompele, 2013; Stadhouders et al., 2010). This will cause a gradient of amplification efficiency between DNA types in competitive PCRs (Bru et al., 2008; Green et al., 2015) and consequently biased sequencing results. Furthermore, within degenerate primer pools, each primer sequence has a different melting temperature and thus amplifies at a different efficiency in PCRs depending on cycling conditions in addition to the increased risk of mis‐priming (Leray & Knowlton, 2017).

This means that even with completely degenerate primer pools, taxonomic biases can only be reduced but not circumvented. There is, however, the possibility to minimize such problems by incorporating a universal tail at the 5′ end of the primers which is not complementary to the template (Green et al., 2015). This approach has been used for conventional barcoding, where the incorporation of, for example, m13 tails in primers can reduce the sequencing of nonspecifically bound primers and boost sequence quality (Ivanova et al. 2007). Using such tailed primers, the PCR can be divided into two steps, where the first few cycles of the PCR (step I) allow a mix of primers of different fit to anneal to matching templates, while the universal tail remains unbound. Later, in the exponential buildup of PCR product (step II), the tail incorporated into the amplicons generated during the first few cycles, now serves as an identical priming site across all templates.

Besides reducing the potential for unequal DNA amplification, these tails have the additional benefit that they facilitate an easy attachment of sequencing adapters and multiplex identifiers (MIDs) needed for NGS. For example, for amplicon sequencing on the MiSeq or HiSeq platforms (Illumina, San Diego, CA, USA), this is commonly done by incorporating a Nextera (Illumina)‐specific tail into locus‐specific primers. This tail is later used by commercially available Nextera indexing kits for tagmentation of samples during re‐amplification of adapters. As this is performed in two separate PCRs, labor costs and contamination risks caused by additional handling increase. However, as an alternative to this approach, it may be possible to perform both amplification and re‐amplification steps in a single PCR, instead of two separate PCRs. This could be done in a PCR comprising two types of cycles (from here on referred to as “single‐tube PCR,” Box 1). This requires that tails that are significantly shorter than the Nextera tails are used, in order to allow tails to have lower annealing temperatures than the locus‐specific primers. Using these tails and by adapting cycling conditions as described by Clarke et al. (2014), it should theoretically be possible to perform both amplification and re‐amplification steps in a single PCR. This single‐tube PCR approach would, if effective, have considerable advantages over traditional NGS library preparation procedures. Most importantly, it could not only significantly reduce the biases from differential amplification of templates during PCR, but also reduce contamination risks and labor as there would be no need for repeated PCRs and handling.

Box 1 **Flowchart of the single‐tube library preparation procedure**

1

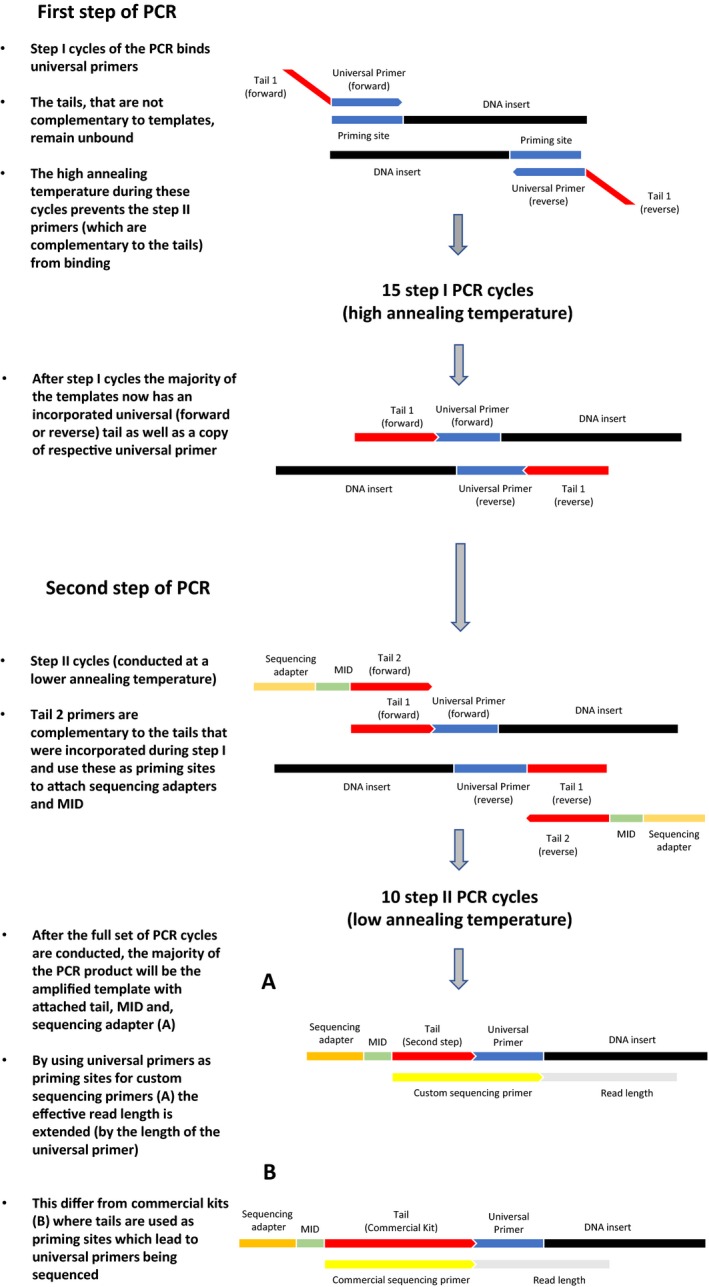

There are three aims for this study:

1. To characterize how commonly used barcoding primers differ in amplification success across all taxonomic animal groups where sequences are available in public databases. Both in order to shed light on, in particular terrestrial, taxa that may have been missed in previous studies investigating published primers, and to aid scientists in selecting appropriate primers for future studies.
2. To test whether it is possible to increase the fit of the best of these primers and thereby reduce biases caused by a lack in conserved priming sites within COI. Specifically, we aim to test whether the amplification of DNA is sufficiently stable across taxonomic groups to allow the COI gene, with its large number of published sequences, to be used for metabarcoding purposes.
3. To equip designed primers with a universal tail and optimize a single‐tube PCR protocol to achieve sequencing results comparable to commercially available kits such as the Nextera indexing kit. The rationale behind this being that such a method would not only counteract biases associated with mixed primer pools but also enable a cost‐ and labor‐effective way of incorporating MIDs and NGS‐specific adapters with a minimized risk of cross‐contamination.

## 2 MATERIAL AND METHODS

### 2.1 Generation of a reference sequence alignment of the COI barcoding region

Using the search criteria “metazoa AND COI” (including alternative dictions for COI), we retrieved all of the sequences available for the barcoding region of COI (~1.3M sequences; May 2015) from NCBI (www.ncbi.nlm.nih.gov/genbank). In order to find conserved regions and compare priming sites located within this gene, it was necessary to align all of these sequences. As creating such a large alignment of sequences would be very computationally demanding, we first reduced this material to include only one (the longest) representative of each unique species. The reduction to only one representative sequence per species was done to eliminate the problem that uneven numbers of representative sequences are available for different taxa. If all sequences would have been kept for the analysis, there would have been the risk that the evaluation of primer fit would be biased toward species with high numbers of representative sequences. This gave a total of ~170,000 unique sequences from throughout the animal kingdom which were aligned using MAFFT 7.271 (Katoh & Standley, 2013). From within this alignment, the “DNA barcoding fragment” was identified and annotated.

### 2.2 In silico evaluation of published primers

**Figure 1:**
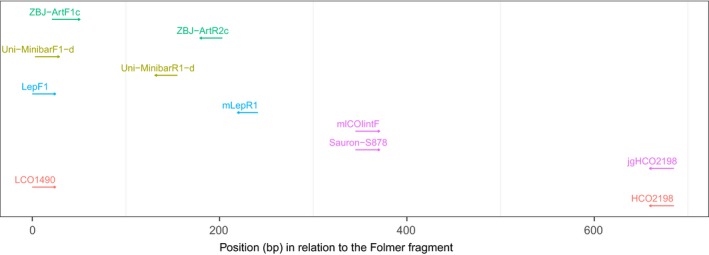
Position of each of the tested primers in relation to the position of the Folmer primers (LCO1490 and HCO2198). Direction (3′ end) of each primer is indicated by arrows, and colors indicate primer pairs tested together

First, we conducted a literature search to investigate which universal primers have so far been used for metabarcoding purposes and selected those that to date had been used most frequently (Table 1). It should here be noted that most of these primers were not designed with complete universality in mind, even though they often have been used in later studies as if they were. Second, we located the position on which each of these primers fit within the alignment of the reference sequences (Figure 1). Then, for each priming site and primer, we implemented a pattern matching algorithm using Biostrings (Pages, Aboyoun, Gentleman, & DebRoy, 2016) to investigate how the number of matching bases between each primer and individual sequences varies. As amplification depends on both the amount of mismatch between primers and sequences, as well as where this mismatch is located (Stadhouders et al., 2010), we here set up two matching criteria: (a) We allowed either 0, 1, 2, or 3 mismatches within the four bases closest to the 3′ end of the primer and (b) nested within each of these, an additional criterion of either 0, 1, 2, 3, or 4 mismatches within the remaining bases of the primer. For each primer and mismatch combination, we then determined the proportion of sequences that met each criterion from within the reference alignment. This allowed us to observe how the fit of each primer varies between a perfect match to a maximum of 7 mismatching bases (three of which would be located in the first four bases of the 3′ end), when amplification is not expected to occur. Furthermore, we chose to evaluate each primer individually to not be restricted to reference sequences where both priming sites are available. This greatly increased the number of reference sequences analyzed for each primer as DNA sequences often did not cover both priming sites for the general primer pairs under evaluation.

**Table 1:** Primers evaluated and designed for this study

Primer	Sequence	Type	References
Uni‐MinibarF1‐d	TCYACTAATCATAAAGATATTGGYAC	Universal Forward	Jordaens et al. (2013)
Uni‐MinibarR1‐d	AAAATTATAATAAARGCRTGRGC	Universal Reverse	Jordaens et al. (2013)
ZBJ‐ArtF1c	AGATATTGGAACWTTATATTTTATTTTTGG	Universal Forward	Zeale et al. (2011)
ZBJ‐ArtR2c	WACTAATCAATTWCCAAATCCTCC	Universal Reverse	Zeale et al. (2011)
LepF1	ATTCAACCAATCATAAAGATATTGG	Universal Forward	Hebert, Penton, Burns, Janzen, and Hallwachs (2004)
mLepR1	CCTGTTCCAGCTCCATTTT	Universal Reverse	Hajibabaei, Janzen, Burns, Hallwachs, and Hebert (2006)
mlCOIintF	GGWACWGGWTGAACWGTWTAYCCYCC	Universal Forward	Leray et al. (2013)
jgHCO2198	TAIACYTCIGGRTGICCRAARAAYCA	Universal Reverse	Geller et al. (2013)
Sauron‐S878	GGDRCWGGWTGAACWGTWTAYCCNCC	Universal Forward	This study
Sauron‐Tail‐S879	CacctgcttctaaatGGDRCWGGWTGAACWGTWTAYCCNCC	Sauron‐S878+ tail	This study
jgHCO2198‐Tail‐A867	CacttcgactctttacTANACYTCNGGRTGNCCRAARAAYCA	JgHCO2198‐A867+tail	This study
i7‐Tail‐A867	CAAGCAGAAGACGGCATACGAGAT[TAAGGCGA]cacttcgactctttac	MiSeq i7+[MID]+ tail	This study
i5‐Tail‐S879	AATGATACGGCGACCACCGAGATCTACAC[CTCTCTAT]cacctgcttctaaat	MiSeq i5+[MID]+ tail	This study
SEQ‐S879	CACCTGCTTCTAAATGGDRCWGGWTGAACWGTWTAYCCNCC	Sequencing primer (read 1, position 18)	This study
IND‐A867	TGRTTYTTYGGNCAYCCNGARGTNTAGTAAAGAGTCGAAGTG	Indexing primer (position 19)	This study
SEQ‐A867	CACTTCGACTCTTTACTANACYTCNGGRTGNCCRAARAAYCA	Sequencing primer (read 2, position 20)	This study

Note

Lowercase letters in the Sauron‐Tail‐S879 and jgHCO2198‐tail‐A867 primers show the location of universal tails. For the step 2 primers, i5‐Tail‐S879 and i7‐Tail‐A867, MIDs are within brackets, followed by MiSeq i7 and i5 adapters.

### 2.3 In vitro evaluation of published and newly designed primers

A large set of DNA extracts from a wide range of animal taxa is available from current as well as previous projects within our laboratory. These extracts were supplemented with additional material to cover a total of 255 DNA extracts from taxonomic groups ranging from nematodes to vertebrates (Table S1). All sample material was supplied and identified by experts, and, if not already DNA extracted, samples were extracted using a Biosprint96^®^ robotic platform with the Biosprint 96 DNA Blood Kit (Qiagen, Hilden, Germany) according to the manufacturer's recommendations, except that TES buffer (0.1 M TRIS, 10 mM EDTA, 2% SDS; pH 8) was used for lysis and 1× TE buffer for elution. Furthermore, to verify that all reference extracts contained amplifiable DNA, each sample was tested in a PCR with universal or phylum‐specific primers.

To evaluate taxonomic coverage for selected and newly designed (see below) primers (Table 1) under laboratory conditions, a series of PCRs were conducted on each reference DNA extract. PCR conditions were standardized for all primer combinations and performed in a reaction mix containing 1.5 μl DNA extract, 0.5 μl bovine serum albumin (BSA; 10 mg/ml), 1 μl of each primer (10 μM), 1 μl dNTP (2 mM), 2 μl reaction buffer with MgCl_2_ (NEB, Ipswich, US) with an additional 0.48 μl MgCl_2_ (25 mM; NEB) to obtain a final concentration of 3 mM, 0.05 μl of oneTAQ (5 U/μl; NEB), and PCR grade water to adjust the volume to 10 μl. Reactions were performed in a Nexus Mastercycler with cycling conditions set to 2 min at 94°C, 35 cycles of 20 s at 94°C, 30 s at 50°C, 60 s at 68°C, and the final elongation for 3 min at 68°C. Amplification of all PCR products was verified using the QIAxel (Qiagen) capillary electrophoresis system with the software BioCalculator 3.2 (Qiagen, Method: AL320) where a DNA fragment of the expected length and with signal strength of ≥0.07 relative fluorescent units was defined as a successful amplification.

### 2.4 Primer design

Informed by in silico results, we used the consensus sequence of the most variable bases in the least variable priming sites to design a new forward primer called Sauron‐S878. In order to increase the fit across all taxonomic groups, additional degeneracy was incorporated into the primer mCOIintF (Leray et al., 2013) which was optimized for aquatic systems and is a modification of the primer C1‐J‐1859 published by Simon et al. (1994). While similar to this primer, Sauron‐S878 was modified to have an improved fit also for terrestrial species and is intended to be used together with the reverse primer jgHCO2198. JgHCO2198 is a highly degenerated version of the commonly used barcoding primer HCO2198 (Folmer, Black, Hoeh, Lutz, & Vrijenhoek, 1994) which in this study had an overall good performance during both in silico and in vitro testing. The combination of Sauron‐S878 and jgHCO2198 will amplify a fragment of ~313 bp (Figure 1), which is suitable for metabarcoding purposes using available NGS platforms (Pompanon et al., 2012).

As part of the objective of this study was to enable the use of these primers to reliably amplify COI for NGS purposes, we adapted both Sauron‐S878 and jgHCO2198 with individually designed universal 5′ end tails (referred to as “tail 1”) in the primers Sauron‐Tail‐S879 and jgHCO2198‐Tail‐A867 (Table 1). These primers can be used to incorporate additional sequences, such as MIDs, that allow samples to be separated after sequencing, as well as platform‐specific adapters, into the 5′ ends of amplicons. Here, this was done for the MiSeq platform (Illumina) in the complementary linker sequences i5‐Tail‐S879 and i7‐Tail‐A867 (Table 1; referred to as “tail 2”). These universal tails together with the locus‐specific primers also double as a template for a set of custom sequencing primers designed for the MiSeq platform that was shown to be effective in a MiSeq sequencing run (read 1 [position #18]—SEQ‐S879, Index [position #19]—IND‐A867, and read 2 [position #20]—SEQ‐A867; Table 1). One of the advantages with these custom sequencing primers is that by including the universal primers as templates, these are prevented from being sequenced. This also results in a read length that is extended by the length of the primer and thus allows a greater overlap (improved quality) when pairing reads during processing of results compared to the Nextera indexing kit, where the sequencing primer binds solely to the long Nextera linker.

To simplify library preparation, we developed a new protocol for attaching these in one PCR as proposed by Clarke et al. (2014). In this two‐step single‐tube PCR approach (flowchart and graphical presentation of included steps in Box 1), the tail 1's were designed to have a lower annealing temperature than the used locus‐specific primers, as well as a poor fit for all reference sequences. This was done to prevent them from annealing to templates during the first step cycles. During these cycles, when annealing temperature is kept high, amplification occurs using universal primers, leading to the unbound tail 1's being correctly incorporated into the PCR product. During later second step cycles, both forward and reverse universal tail 1's are used as priming sites for tail 2's. During this second step, annealing temperature is lowered in order to allow the tail 2's to bind to tail 1 templates. This activates the step 2 primer and allows amplification to occur on the step one tail, which, after the first step, will be identical across all amplicons.

As the tails may alter the characteristics of original primers, tail‐incorporated primers (Sauron‐Tail‐S879, jgHCO2198‐Tail‐A867) were tested in parallel with selected and designed universal primers during in vitro testing to ensure that this tail did not negatively affect amplification efficiency for any DNA extracts. PCR conditions were optimized by varying primer concentrations, annealing temperature, and cycle numbers between the first and second step of the single‐tube PCR until the majority of the final PCR product was a DNA fragment of the desired length containing platform adapters, MIDs as well as template DNA. We again confirmed that no DNA extracts failed to amplify using this approach where amplification was previously successful and subjected a subset of these extracts to Sanger sequencing to confirm the incorporation of platform adapters and MIDs.

After optimization, the PCR set‐up that produced the strongest products of the targeted fragment with a minimum of unwanted amplification of primer artifacts was a reaction mix of 2 μl DNA extract, 0.5 μg of BSA (10 mg/ml), 0.15 μl of each 1st step primer (10 μM; Sauron‐Tail‐S879, jgHCO2198‐Tail‐A867), 1 μl of each 2nd step primer (10 μM; i5‐Tail‐S879, i7‐Tail‐A867), 5 μl of Multiplex PCR Kit reaction mix (Qiagen), and PCR grade water to adjust the volume to 10 μl. Cycling conditions were set to 15 min at 95°C, 15 1st step cycles of 30 s at 94°C, 90 s at 55°C, 60 s at 72°C, and 20 2nd step cycles of 30 s at 94°C, 90 s at 45°C, 60 s at 72°C, and a final elongation of 10 min at 72°C.

### 2.5 Evaluation of newly designed primers and single‐tube PCR approach for NGS

For the final evaluation, we assembled three pooled mock communities from among the 253 DNA extracts (there was no sufficient volume remaining after in vitro testing of two DNA extracts that had to be excluded) of 84–85 species each (Table S1). To do this, the DNA concentration of each of the reference extracts was first quantified (mean of three individual measurements of different DNA aliquots) using a NanoDrop 1000 (Thermo Scientific, Wilmington, DE, USA). After this, DNA extracts were picked such that each mock sample included a unique mix of extracts representing an equal coverage of taxonomic groups and DNA extract concentrations (Table S1). Each of these mock samples was then further subdivided into (a) an unbalanced pool, where an equal volume of each DNA extract was pooled, and (b) a balanced pool where all DNA extracts with a DNA concentration higher than 5 μg/μl were diluted to 5 μg/μl and then mixed with the remaining extracts (<5 μg/μl DNA) at equal volumes. As the NanoDrop cannot measure low DNA concentrations precisely, the DNA extracts with a concentration below 5 μg/μl were left unaltered, to avoid pretending a higher precision than could be obtained. Each mock sample was replicated three times during the following tests to account for variability in PCRs and NGS. This gave a total of 18 individually tested mock communities (three sets of DNA extracts × 2 balance levels × 3 replicates), each uniquely tagged with MIDs using the developed single‐tube PCR approach (further on referred to as single‐tube mock communities).

### 2.6 Library preparation

Each of the 18 single‐tube mock communities was prepared in 50 μl reactions and combined into a ready to sequence library, by first amplifying these using the developed primers and the single‐tube PCR approach (for details on PCR conditions and protocols, see Data S1). Furthermore, to compare how the single‐tube approach performed in comparison with commercial kits such as the Nextera indexing kit (Illumina), an additional library was prepared that included each three replicates of the three mock communities with balanced concentrations. This second library included a version of each of the LepF1/mLepR1, mCOIintF/jgHCO2198, and Sauron‐S878/jgHCO2198 primer combinations with a Nextera specific tail incorporated at the 5′ end of each primer according to the manufacturer's recommendation (Table 1). Preparation of this library included a first amplification using Nextera tail‐incorporated locus‐specific primers, followed by a first clean up of unwanted primers and artifacts. After this, an additional re‐amplification was performed to incorporate sequencing adapters and MIDs (for details on PCR conditions and protocols see Data S1).

For each library preparation method, amplicons from individual samples with incorporated MIDs and sequencing adapters were cleaned using SPRIselect magnetic beads (Beckman Coulter, Bread, CA, USA) according to the manufacturer's recommendation for left side size selection with a ratio of beads to PCR product volume of 0.8. After clean up, the remaining DNA was quantified using the QIAxel (Qiagen) capillary electrophoresis system with the software ScreenGel v1.4 (Qiagen). Thereafter, all samples were pooled at equimolar concentrations into ready to sequence libraries to ensure an even sequencing depth across samples. Ready‐to‐sequence libraries were submitted along with sequencing primers for paired‐end sequencing on an Illumina MiSeq platform using the MiSeq Reagent Kit Nano v2 (300 bp; Illumina) at the Biomedical Sequencing Facility of the CeMM Research Center for Molecular Medicine of the Austrian Academy of Sciences and the Medical University of Vienna.

### 2.7 Data analyses

Raw sequencing reads were demultiplexed, quality‐checked, trimmed, and combined into paired‐end reads using Usearch (Edgar, 2010). To remove all singleton sequences and reads shorter than 300 bp, these reads were then dereplicated using Usearch. After this, remaining reads were clustered into OTUs based on a 97% sequence similarity using Usearch. From among clusters, the centroid sequence was selected as a representative and a taxonomic ID was assigned using blastn (Altschul, Gish, Miller, Myers, & Lipman, 1990) based on the NCBI Nucleotide database (Benson, Karsch‐Mizrachi, Lipman, Ostell, & Wheeler, 2006) with a minimum ID threshold set to 90% and a word length of 28 bases. From received hits, the most likely identity was then selected based on E‐score (with a maximum threshold of e^−10^), match length, and on known information about the expected identity of the included taxa.

Because of the reduced overlap caused by the sequencing of the universal primers from Nextera libraries with the commercial sequencing primers (Box 1), as well as slightly different phred scores between runs (single‐tube library, 27.3_mean_, ±2.6_*SD*_; Nextera, 35.7_mean_, ±1.9_*SD*_), pairing of reads from the Nextera sequencing run needed to be more restrictive. In order to make results comparable between the two libraries, we first analyzed the single‐tube results for each replicate of the three balanced concentration mock samples using the same setting as for the Nextera library preparation method. Then, after reads from the two library preparation methods had been compared, the single‐tube library reads were reprocessed by allowing more mismatches during pairing due to the increased overlap in these reads in order to increase the number of paired reads that passed quality filtering. The reason for doing this was here to make it easier to interpret whether any potential differences in libraries were due to the artifacts of the preparation method or due to a difference in overlap between forward and reverse reads between methods.

### 2.8 Statistical analysis

All analyses were based on the presence or absence of in silico or in vitro amplification, and statistical tests were performed using generalized linear models fitted with binomial error distributions. For each model, diagnostic plots were examined to confirm that model assumptions were met (Zuur et al. 2010). NGS analyses were based on the presence or absence of detections, results from mock communities were combined as each mock community contained a different set of taxa, and replicates were used to test whether the successful detection of an extract varies between taxonomic classes. To model the probability of detecting an included DNA extract given the concentration of the DNA of this taxon in relationship to the concentration of other extracts, and how this probability is affected by the number of sequences attained (sequencing depth), values were predicted from binomial models fitted with measured data. All statistical tests were performed in R version 3.3.2 (R core team, 2016).

## 3 RESULTS

### 3.1 In silico

**Figure 2:**
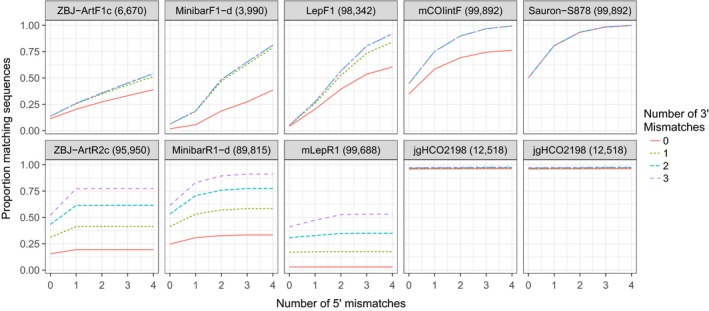
Overall proportion of NCBI sequences matching the 3′ region (first four bases; line type) and 5′ region (rest of the primer; *x*‐axis) of general COI barcoding primers. Forward and reverse primers are on top of each other, and the number of NCBI sequences on which each primer was tested is shown in parentheses beside the primer name. The newly designed forward primer (Sauron‐S878) is on the right‐hand side and combined with jgHCO2198, as was the primer mCOIintF. Note that for Sauron‐S878, mCOIintF, and jgHCO2198, there is almost no variability in fit for the 3′ end and lines are therefore overlapping

**Figure 3:**
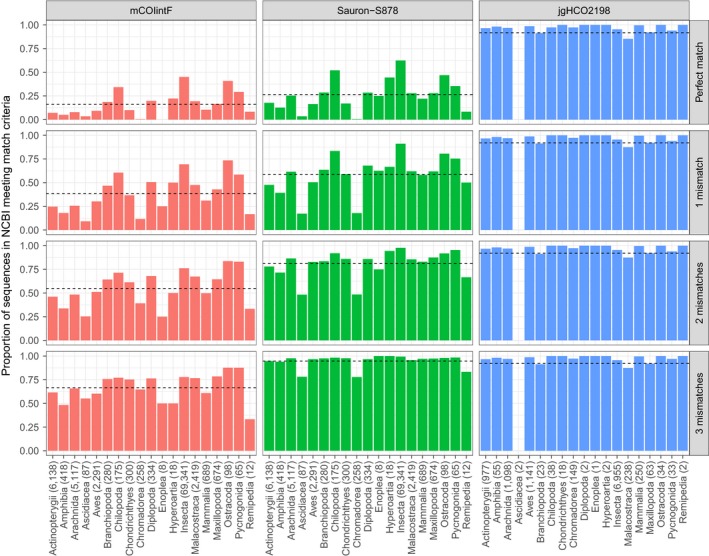
In silico testing. Proportion of NCBI sequences matching (full match in the first four bases of the 3′ end and 0–3 mismatches in the remaining 5′ region; figure rows) general COI primers (figure columns) for different classes of animals with the number of NCBI sequences covering the priming site in parentheses. The newly designed forward primer (Sauron‐S878) is in the central column flanked by mCOIintF (left) and the reverse primer jgHCO2198 (right). Horizontal dashed lines show mean coverage of all NCBI sequences

In silico evaluation of priming site variability showed that the newly designed forward primer Sauron‐S878, in combination with the reverse primer jgHCO2198, performed better than all other primer pairs (*p* < 0.001). In fact, most of the tested metabarcoding primers exhibited considerable variation in both number and position of mismatches between primers and sequences. Among published primers, all but the reverse primer jgHCO2198 had a variable fit across taxonomic groups in the 3′ end (Figure 2) where a good fit is necessary for amplification to take place. Under restrictive conditions (no mismatches allowed in the 3′ end of the primer and three mismatches between the remainder of the primer and template), the best forward primer (*p* < 0.001) was our new primer Sauron‐S878 that fit on 98.3% of NCBI sequences (Figure 3). The next best fitting forward primer mCOIintF fit only on 74.4% of the same sequences due to a mismatch at the 3′ end. Taxonomic groups that were fitting poorly with mCOIintF were, for example, Ascidiacea (65.5%), but also common groups such as Arachnida (65.8%), nematodes (64.7%), and many vertebrates: Mammalia (61.0%), Aves (60.3%), Amphibia (48.3%), and Actinopterygii (61.6%). For each of these groups, the new primer Sauron‐S878 considerably increased coverage to 78.2% for Ascidiacea, 97.6% for Arachnida, 77.9% for nematodes, 97.4% for Mammalia, 96.7% for Aves, 94.0% for Amphibia, and 94.8% for Actinopterygii.

Among the reverse primers, only jgHCO2198 had an overall good fit of 96.2% and only performed poorly for Ascidiacea which were missed completely (although it should be noted that a reference sequence for the jgHCO2198 binding site was only available for two species within this group). With a more relaxed fit criterion (seven mismatching bases of which three are located in the first four bases of the 3′ end), when the least well‐fitting of the primers will not amplify and a considerable bias is expected, the best fitting of the published primers continued to be the primers mCOIintF and jgHCO2198. Furthermore, even if individual forward or reverse primers among other primers sometimes performed well, it should be noted that under these conditions, the least well‐fitting primer of each primer pair (forward/reverse) only found a match on 81.4% (MinibarF1‐d), 54.4% (ZBJ‐ArtF1c), and 53.1% (mLepR1) of sequences.

### 3.2 In vitro

**Figure 4:**
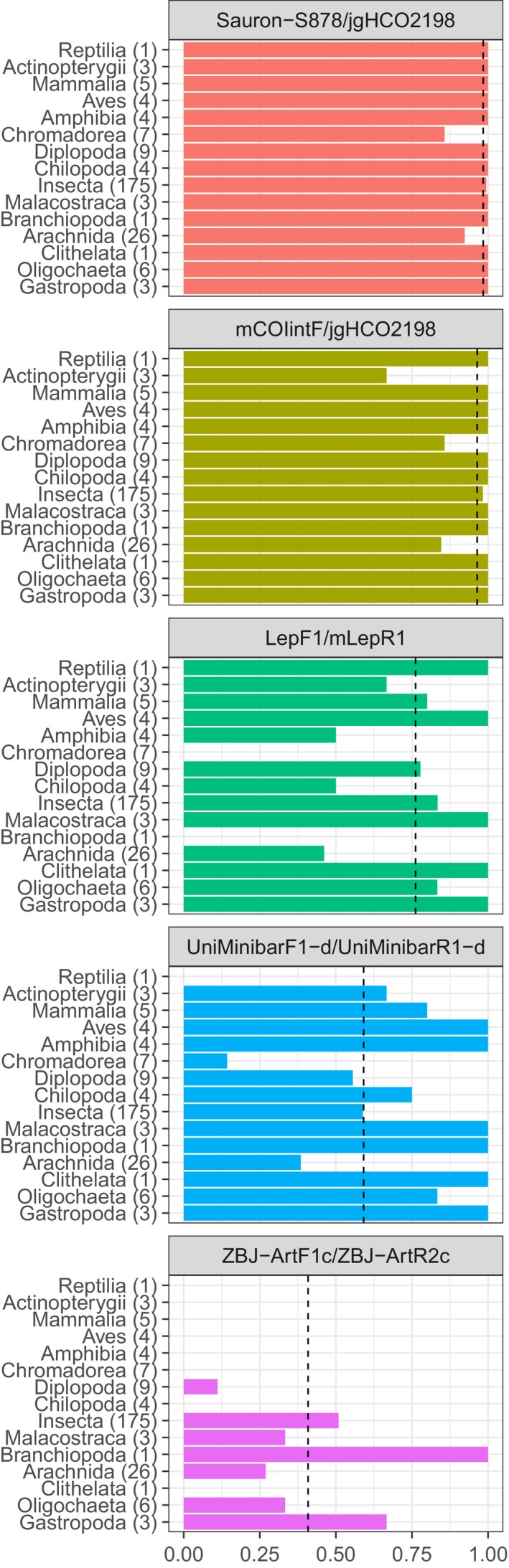
In vitro testing. Proportion of DNA extracts amplified from each taxonomic group (number of tested individuals in parentheses) with commonly used “universal” primer pairs ranked by high (top) to low (bottom) amplification success. The newly designed primer Sauron‐S878 is here located at the top as the primer with the highest amplification success. Dashed vertical lines show mean detection success

In in vitro testing, the newly designed primer Sauron‐S878 combined with the reverse primer jgHCO2198 had an amplification success of 98.4%, and continued to perform significantly better than other primer pairs (Figure 4; *p* < 0.001), with the exception of mCOIintF/jgHCO2198 that also performed well with an amplification success of 96.4%. Of the other primer combinations, LepF1/mLepR1 amplified 76.2%, MinibarF1‐d/MinibarR1‐d 59.1%, and ZBJ‐ArtF1c/ZBJ‐ArtR2c 40.9% of the tested 255 taxa. While mCOIntF/jgHC2198 did have an overall good amplification efficiency, it did perform poorly for Arachnids (84.6%), where common groups such as Linyphiidae did not always amplify, for Actinopterygii (66.6%), and for nematodes (85.7%). While neither of these groups were statistically different from other groups amplified by the same primer, we can here show that by replacing the primer mCOIintF by the new primer Sauron‐S878, the amplification success of these groups could be increased, to 92.3% for Arachnids, 100% for Actinopterygii, and only the amplification of nematodes remained unchanged at 85.7%.

### 3.3 NGS results

**Figure 5:**
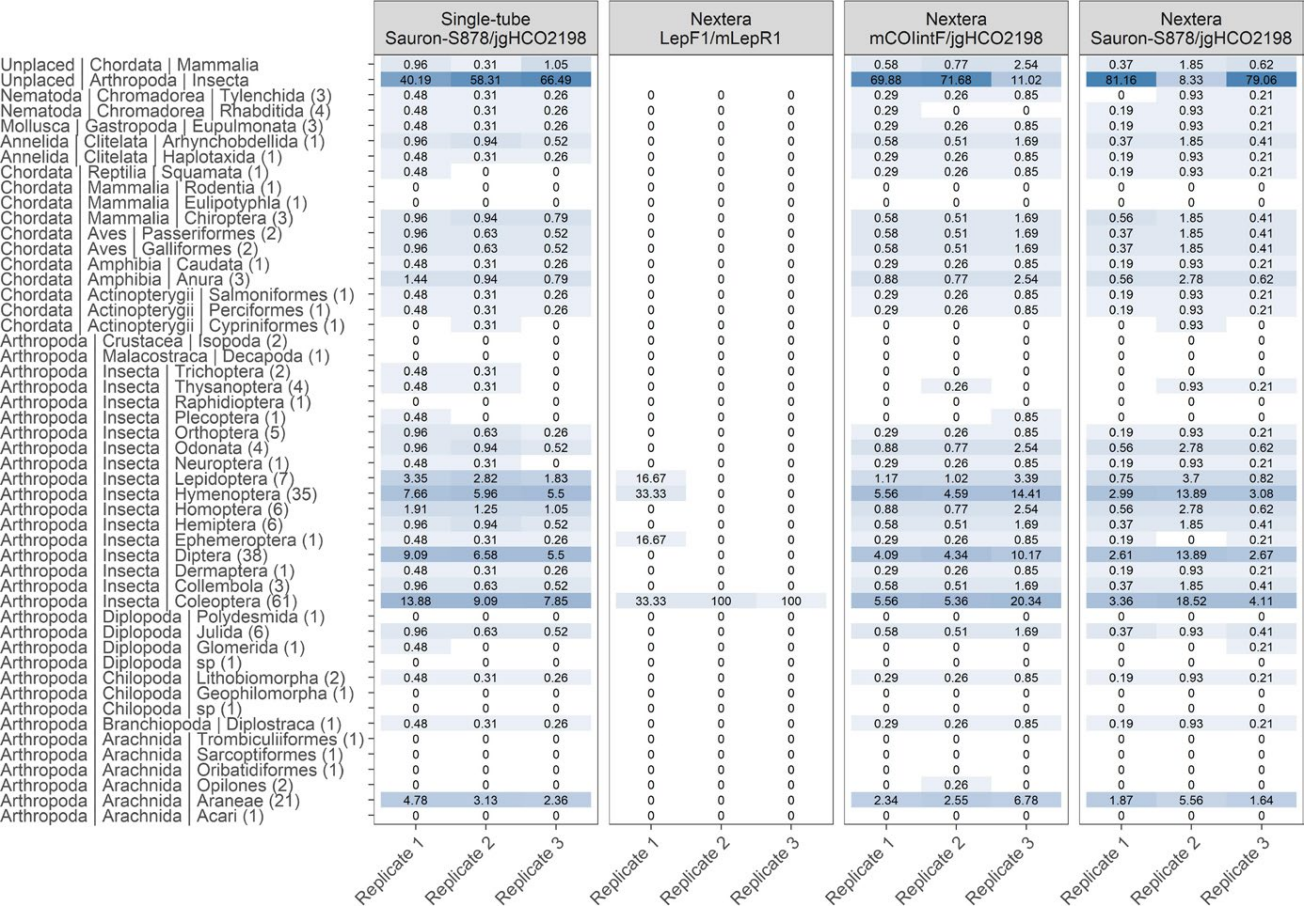
Percentage of retrieved samples from taxonomic orders from balanced, replicated mock communities (for simplification all three mock communities are pooled) by primer pair and library preparation method. Color is proportional to the relative number of retrieved sequences

From the library prepared using the developed single‐tube PCR approach and custom sequencing primers, a total number of 607,528 raw paired‐end reads were generated from a small run on the MiSeq platform. After preprocessing, these ranged from 27,507 to 55,094 reads distributed between balanced and unbalanced, replicated mock samples with a mean read number of 36,637. To test how the developed single‐tube PCR approach and custom sequencing primers perform against the commercially available Nextera indexing kit (Illumina), a second small run was conducted on the same MiSeq platform. From this run, 546,099 raw paired‐end reads were generated that, after preprocessing, ranged from 10,035 to 30,729 reads distributed between balanced, replicated mock samples and primers with a mean read number of 20,226. These reads were subsequently compared to the 277,778 raw paired‐end reads generated from the balanced mock samples included in the first MiSeq run (single‐tube PCR approach). After processing and matching of the sequences produced by the Nextera library to the NCBI Nucleotide database, the proportion of individual samples in each mock community that could be identified using each primer combination (Figure 5) was considerably lower for the LepF1/mLepR1 primer combination (2%, *p* < 0.001), than for mCOIintF/jgHCO2198 (42%), and Sauron‐S878/jgHCO2198 (40%). From the Nextera library, samples amplified by the latter two primer combinations showed no differences. However, compared to the Nextera preparation method, the single‐tube PCR approach in combination with the Sauron‐S878/jgHCO2198 primers showed an increased recovery rate of included taxa of 50% (*p* < 0.05). Furthermore, it was also noticeable that in most replicates, the single‐tube method produced less sequences from arthropods that were not included in mock samples than the Nextera method (unplaced arthropods, Figure 5). This suggests that the extra overlap between reads with the single‐tube method may decrease read errors even though these still occur. This was also indicated when the sequence database was reduced to include only those species included in mock samples. This unreported test is biased as reads are forced to align to sequences, but nevertheless allowed the proportion of taxa recovered to be increased to ~68% with the single‐tube method.

**Figure 6:**
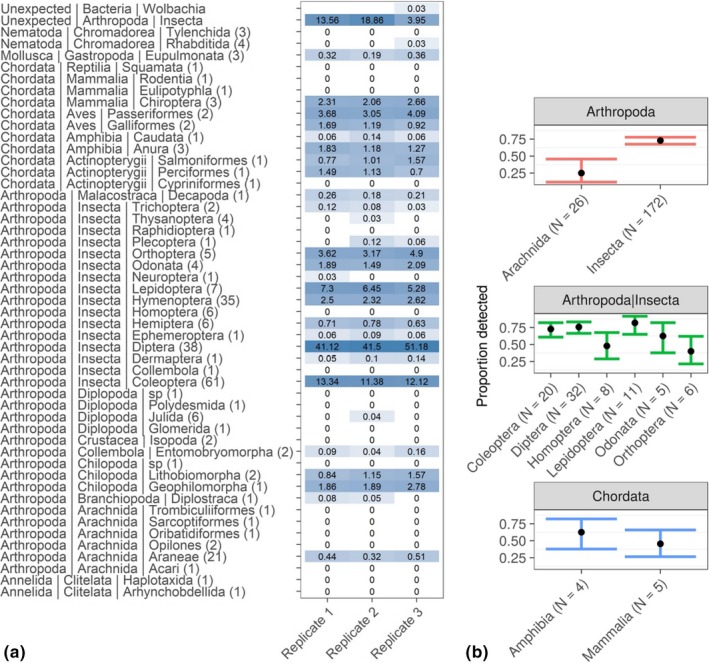
(a) Percentage of retrieved sequences per sample from taxonomic orders (number of included samples in parentheses) from balanced, replicated mock samples (for simplification all three mock communities are pooled). Color is proportional to the relative number of retrieved sequences. For ease of visualization, samples have been grouped taxonomically, for a complete list of detected taxa, see Table S1 (Supporting Information). (b) Variability in the proportion of extracts identified with NGS from mock communities (*y*‐axis) per taxonomic group (*x*‐axis). Error bars represent 95% confidence intervals, and sample sizes are shown within parentheses

After reads from the two library preparation methods had been compared, we found that because of the increased overlap between the single‐tube library reads, these could be better processed by allowing more mismatches during pairing of reads. Thus, we reanalyzed this library and again matched processed reads against the NCBI Nucleotide database. The proportion of individual samples in each mock community that could be identified among the samples included at balanced concentrations now varied between 87% and 67%, and in total 75% of the DNA extracts. Among the DNA extracts that did not produce sequences were several underrepresented taxonomic orders (Figure 6a; for a detailed list of identified taxa, see Table S1). However, after correcting for false discovery rate, a significant bias could only be found among well‐represented groups (where there were more than four samples for statistical relevance; Figure 6b) for arachnids (*p* < 0.001). It should be noted that among the included DNA extracts that produced no sequences, all missed arachnids had a low DNA concentration (<2 μg/μl).

**Figure 7:**
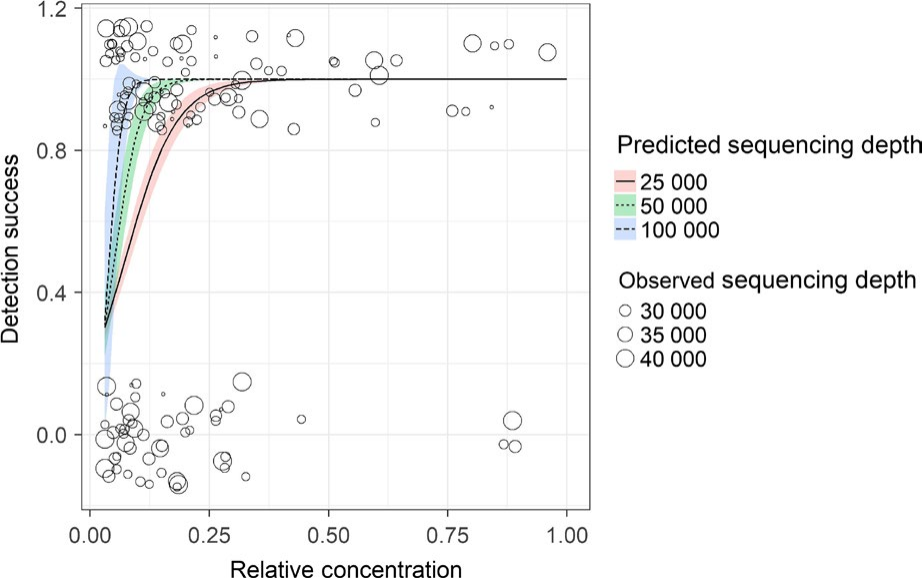
Proportion of extracts identified with NGS from mock communities (*y*‐axis) modeled against the proportion of sample DNA concentration in relation to total DNA concentration (*x*‐axis). Colors show the influence of different levels of sequencing depth and depict the predicted standard error. Observed data are here shown by circles proportional in size to sequencing depth

From unbalanced mock samples, we could show that these dropouts may alternatively be attributable to an insufficient sequencing depth. Particularly, we could see that the lower the proportional DNA concentration of a species was within a sample, the more sequences needed to be read to reliably recover this species from within meta‐samples. We modeled the likelihood of detecting individual sample sequences within each mixed sample, against the proportion of a sample's DNA concentration in relationship to the total DNA concentration (before PCR), and showed that the likelihood of detection of low concentration samples drops considerably, particularly at lower sequencing depth (Figure 7, *p* < 0.01, _McFadden_ *R* ^2^ = 0.25). This relationship was additionally reflected in the DNA extracts with a concentration of <5 μg/μl that were detected at a lower rate (*p* < 0.001) in balanced mock communities (32%, *SE* = 0.11) than higher concentration extracts (69%, *SE* = 0.14) included at equimolar proportions.

## 4 DISCUSSION

In this study, we showed that, while the concerns about using COI in metabarcoding studies have been growing (Clarke et al., 2014; Deagle et al., 2014; Leray et al., 2013), new primers such as Sauron‐S878, mCOIintF, and jgHCO2198 make it possible to, even though species sometimes are missed, reliably amplify COI from a wide range of taxa. We did so by extensively testing these primers in vitro on DNA and in silico on sequences from a large set of taxa. In addition, primers were adapted and tested with a universal tail that, within a new single‐tube PCR laboratory protocol, allows NGS‐specific adapters to be incorporated in a single PCR where previously additional laboratory procedures were needed. As additional benefit, the effective read length and thus sequence overlap of paired‐end reads can be extended with this approach compared to the commercial Nextera indexing kit. Using this methodology, we show how NGS libraries can be prepared as efficiently as with commercial kits but with a reduction in PCR‐related biases from primer mismatches, handling as well as a reduction in the work normally needed to attach sequencing adapters and MIDs.

### 4.1 Primer comparison

We demonstrated that many of the so far published primers do exhibit a large amount of bias across taxonomic groups during both in silico and in vitro testing which should be considered when selecting primers and interpreting results. Because of the variability in COI, the primers may miss some groups completely and have poor amplification efficiency in other taxa. The best results during in vitro and in silico testing were obtained with the primer pair Sauron‐S878/jgHCO2198 which matched and amplified over 98% of the evaluated taxa. The next best option was the combination mCOIintF/jgHCO2198 which performed also well during the in vitro testing, but matched only 74.4% of the taxa in NCBI due to a poor fit on important terrestrial taxa such as spiders, but also many vertebrates. By incorporating additional degeneracy into the primer Sauron‐S878 in order to better cover terrestrial groups such as spiders, we also improved the fit for a large range of other taxa where a mismatch situation was occurring at the 3rd base from the 3′ end. This improved fit suggests that in a competitive reaction, fewer taxa are likely to be amplified with low efficiency due to poor primer fit, reducing the overall primer bias.

All other primers failed to amplify large numbers of the tested DNA extracts: for example, ZBJ‐ArtF1c/ZBJ‐ArtR2c failed to amplify over half of the tested DNA extracts. Such biases are problematic, particularly if one is unaware of them. On the other hand, under certain circumstances, this can also be beneficial. For example, in the trophic studies on vertebrates, these primers mostly have been used for, one is usually not interested in amplifying consumer DNA. In such cases, primers that do not (or poorly) amplify consumer DNA might be advantageous (Deagle et al., 2009), although this means accepting a high degree of bias in the detection of prey taxa, especially as the primers ZBJ‐ArtF1c/ZBJ‐ArtR2c not only missed vertebrate DNA but also many taxa of arthropods.

### 4.2 Single‐tube protocol and NGS

To maximize taxonomic coverage, Sauron‐S878 is a highly degenerate primer being in the end a mixture of 768 different primer combinations. To compensate for the potential problems caused by differing annealing temperatures between primers known to occur with such a high degree of degeneracy as in both Sauron‐S878 and jgHCO2198, we incorporated universal tails into these primers (Green et al., 2015). The rationale here was that primer bias is likely to be reduced by conducting the majority of the exponential PCR cycles with these tails as identical templates across amplicons. We here, in addition, developed an approach that uses this tail to split a single PCR into two steps where (a) a first step of amplification is conducted using the universal primer pair at a higher annealing temperature to obtain higher specificity for the priming site and (b) a second step amplification is conducted using the universal tails as templates at lowered annealing temperature. This approach not only has the potential to reduce biases associated with high degrees of degeneracy, but can be used to cost‐effectively include NGS adapters into amplicons for library preparation.

The developed single‐tube PCR approach was used in combination with a set of custom sequencing primers and was confirmed to work well compared to the standard Illumina primers used with the Nextera library preparation method on a MiSeq run. An additional benefit of the single‐tube PCR approach is that, unlike with the Nextera preparation method, this method uses the locus‐specific primers as part of the template for sequencing primers, meaning that these are not sequenced. This allows a longer insert length, which facilitates pairing of reads as the overlap between forward and reverse reads is extended by the length of each primer. For example, in our case, the extended read length achieved with the single‐tube approach allowed considerably more taxa to be recovered from within the tested samples, as fewer reads had to be discarded due to poor quality scores.

To test for biases between taxonomic groups, we used the single‐tube PCR approach to prepare a library of three replicated mock samples constructed from the DNA extracts used during primer testing. We did not detect any significant bias between the included taxonomic groups, even though not all of the included DNA extracts did produce sequences. It should, however, be noted that it had been confirmed that these DNA extracts did amplify with the primers using the developed protocol during in vitro testing. From the subset of the mock samples where initial extract concentrations had not been balanced for concentrations, we could see a likely explanation for this: The more uneven DNA concentrations are within a sample, the more sequences need to be read to reliably recover lower concentration samples from within mixed samples. More specifically, the probability of detecting a species will depend on both the relative concentration of the DNA of a given species in relationship to other species in the same sample as well as sequencing depth. This bias in combination with potential primer biases may lead to more or less random numbers of reads obtained for different taxa from an individual NGS run. Other studies have here come to similar conclusions, and it has repeatedly been stressed that when interpreting the results from metabarcoding studies, the detection of taxa should preferably be treated as the presence or absence as the number of returned sequences may be misleading (Elbrecht & Leese, 2015; Ficetola et al., 2015; Yu et al., 2012). Here, we would like to suggest that this argument may be overly simplified and suggest that even the presence/absence data may carry a considerable bias if the proportion of DNA types is uneven between mixed samples and sequencing depth is low as sampling may not be complete. Consequently, as the relative DNA concentration in most cases is unknown and not only affected by the amount of tissue from which the DNA is extracted, but also by the level of DNA degradation, tissue type and copy number (Deagle, Eveson, & Jarman, 2006; Thomas, Deagle, Eveson, Harsch, & Trites, 2015), care should be taken when deciding how many samples to pool in one run. The comparably low sequencing depth (10,000–30,000 quality‐checked reads for each sample with 85 taxa to be identified) in this study was chosen, to mimic the suggestion to sequence hundreds or thousands of samples in one run making use of a nested tagging approach (Kitson et al., 2015). The idea behind this is that few thousand sequences per sample would be sufficient in obtaining the desired information and everything else would be lost investment. However, we could clearly show that with such a reduced sequencing depth, the risk to miss rare taxa increases dramatically. The basic assumption that NGS will describe all of the sequences that are in a sample is simply not true, but what is more likely to occur is that one describes the most abundant DNA types in a sample unless sequencing depth is sufficiently high. As the abundance of DNA types in a sample is not necessarily related to the most abundant species in an environment, or diet, there is definitely a need for caution in interpreting metabarcoding results.

## 5 CONCLUSIONS

The Sauron‐S878 and jgHCO2198 primers enable researchers to continue employing COI for metabarcoding purposes without considerable primer biases in order to make use of the extensive available sequence databases. The developed protocols can reduce potential amplification biases produced by the (necessary) high degree of degeneration, such as preferential amplification depending on annealing temperature. Furthermore, the single‐tube PCR approach for library preparation minimizes the contamination risk due to repeated handling and re‐amplification. For metabarcoding results, however, we would argue that there are still additional methodological biases present that so far have not been well addressed but may strongly affect the completeness of results. Because of this, scientists need to consider carefully how much sequencing depth is needed for describing DNA from mixed samples. Mainly because the relative proportions of DNA from different species in such samples are usually not known but drastically affect detection probability, especially for DNA contained in low proportions.

Finally, it should be mentioned that most of the biases discussed in this paper are exponentially magnified by the current need to conduct PCRs before sequencing. Without exponential amplification in PCR, differences in detection probability are likely to be smaller. However, even though PCR induced biases will eventually be circumvented by PCR‐free approaches (Creer et al., 2016; Denver et al., 2016; Paula et al., 2016), more abundant DNA will still be preferentially described from mixed samples.

## AUTHOR CONTRIBUTIONS

MT and DS obtained funding and together with OR conceived/designed the study. DS and NH performed laboratory work. OR conducted all analyzes of data, interpreted results, and compiled tables and figures. OR wrote the first draft of the manuscript, and MT and DS contributed to finalizing the paper. All authors gave final approval for publication.

## DATA ACCESSIBILITY

The data generated for this study have been deposited in the Dryad Repository, https://doi.org/10.5061/dryad.5jq5t36.
