# Supplementary material for: A broadly applicable COI primer pair and an efficient single‐tube amplicon library preparation protocol for metabarcoding
Source: Ecol Evol. 2018 Dec 11;8(24):12335–50. doi: 10.1002/ece3.4520 (PMC6308894; doi:10.1002/ece3.4520)
Supplement: Supplementary file 2 [file ECE3-8-12335-s002.docx]

**Data S1**

Laboratory procedures for preparation of the single-tube libraries in 50 µl reaction volumes

Using the single tube approach, each PCR was performed in a reaction mix containing 15 µl DNA extract, 2.5 µl of bovine serum albumin (BSA; 10 mg/ml), 0.75 µl of each 1^st^ step primer (10 µM), 2.5 µl of each 2^nd^ step primer (20 µM), 25 µl of reaction mix (Multiplex PCR Kit Qiagen) and 1 µl PCR grade water to adjust the volume to 50 µl*.* Cycling conditions in a Mastercycler Nexus (Eppendorf, Germany)were set to 15 min at 95 °C, 15 step I cycles of 30s at 94 °C, 90s at 55 °C, 60s at 72 °C, and 20 step II cycles of 30s at 94 °C, 90s at 45 °C, 60s at 72 °C, and a final elongation of 10 min at 72 °C.

Laboratory procedures for preparation of the Nextera library

1^st^ PCR: Amplification with locus specific primers

Individual PCRs of samples were performed in a reaction mix containing 15 µl DNA extract, 2.5 µl bovine serum albumin (BSA; 10 mg/ml), 2.5 µl of each primer (10 µM), 25 µl Qiagen reaction mix (Multiplex PCR Kit Qiagen), and PCR grade water to adjust the volume to 50 µl. Reactions were performed in an Eppendorf Mastercycler with cycling conditions set to 15 min at 95 °C, 25 cycles of 30s at 94 °C, 90s at 50 °C, 60s at 72 °C and a final elongation of 10 min at 72 °C.

1^st^ PCR cleanup using Exosap

PCR products were after 1^st^ PCR cleaned with the ExoTSAP procedure using 0.1 units of TSAP, and 0.1 units of EXO to each 10µl of PCR product. Samples were incubated at 37 °C for 15 min which was followed by a heat inactivation step at 80 °C for another 15 min following the manufacturers recomendations.

2^nd^ PCR: Re-amplification to attach Nextera indexes

Reamplification PCRs of samples were performed in a reaction mix containing 15 µl cleaned 1^st^ PCR product, 2.5 µl bovine serum albumin (BSA; 10 mg/ml), 2.5 µl of each indexing primer (10 µM), 25 µl Qiagen reaction mix (Multiplex PCR Kit Qiagen), and PCR grade water to adjust the volume to 50 µl. Reactions were performed in an Eppendorf Mastercycler with cycling conditions set to 15 min at 95 °C, 8 cycles of 30s at 94 °C, 90s at 55 °C, 60s at 72 °C and a final elongation of 10 min at 72 °C.
